# Swallowing Apraxia Post Ischemic Stroke

**DOI:** 10.3390/ijerph192316329

**Published:** 2022-12-06

**Authors:** Abdullah Mohammed Alfaris, Atheer Saeed Alghamdi, Enas Saad Almowalad, Awad Aweid Al Harbi, Khaled Abdulraheem Alghamdi, Jameelah Saeedi, Nisreen Naser Al Awaji

**Affiliations:** 1 Rehabilitation Department, King Abdullah Bin Abdulaziz University Hospital, P.O. Box 47330, Riyadh 11552, Saudi Arabia; 2 Department of Internal Medicine and Critical Care Neuroscience Division, King Abdullah Bin Abdulaziz University Hospital, P.O. Box 47330, Riyadh 11552, Saudi Arabia; 3 Department of Health Communication Sciences, College of Health and Rehabilitation Sciences, Princess Nourah Bint Abdulrahman University, P.O. Box 84428, Riyadh 11671, Saudi Arabia

**Keywords:** swallowing apraxia, ischemic stroke, VFSS, FEES

## Abstract

A 55-year-old male patient with a known medical history of diabetes mellitus type 2 and treated lymphoma was first admitted with a sudden left-sided facial asymmetry and mouth deviation to the left side with no other neurological symptoms. A Computerized Tomography (CT) scan of the brain showed acute infarct and small left basal ganglia old lacunar infarction. He was discharged on a dual antiplatelet. One week later, the patient’s condition had worsened and, therefore, was admitted with an impression of ischemic stroke. A bedside swallowing assessment, VFSS, and FEES study were conducted to diagnose this case. The bedside assessment did not reveal any sensory or motor deficits in his oral cavity and the FEES examination was also unable to rule out pharyngeal dysphagia. However, a videofluoroscopic swallowing study (VFSS) revealed a significant dysfunction of oral preparation and oral phases and presented difficulty initiating the pharyngeal phase. Given these features, we believe that this swallowing difficulty is caused by swallowing apraxia. This case provides additional information and understanding on management from the swallowing side.

## 1. Introduction

Dysphagia is a common impairment following hemorrhagic and ischemic stroke related to cortical and subcortical lesions. Approximately 50% of acute stroke survivors present with dysphagia [1,2]. With some research reporting percentages as high as 81% [3], apraxia is a neurological disorder of learned purposive movement skills that cannot be explained by deficits in elemental motor or sensory systems. This definition of apraxia sometimes refers to disorders involving volitional movement with preserved nonvolitional movement. Apraxia includes a range of neurological, acquired, and developmental disorders, such as buccofacial, gaze, limb, speech, and swallowing [4]. Swallowing apraxia may occur after a stroke [5]. The unique characteristics of this type of swallowing dysfunction, which Robbins and Levine [6] attribute to a “lack of coordination of labial, lingual, and mandibular movement during the oral stage of swallowing” (p. 14), include delayed initiation of bolus transfer, with no lingual movement and lingual searching motions before initiating oral transfer, uncoordinated lip, tongue, and mandible movements, and no sensory impairment or dyskinesia [7]. Swallowing apraxia poses a challenge to speech—language pathologists.

Normal swallowing involves both voluntary and involuntary physiologic components [8]. Swallowing proceeds from more-to-less volition as the bolus moves from the oral stage to the pharyngeal stage [5]. The oral stage of swallowing is volitional and therefore predominantly controlled by the cortex and the pharyngeal stage is primarily involuntary and controlled by the integration of supratentorial regions with the brainstem; the automatic swallowing function is largely preserved in the swallowing apraxia [5].

Although apraxia has been discussed in detail in the literature (e.g., [2,4,8]), there are fewer studies on swallowing apraxia (e.g., [5,7]). However, the literature on swallowing apraxia has improved the overall understanding of this disorder and improved its diagnosis and case management. In this report, we describe an interesting case of swallowing apraxia after an ischemic stroke and case management by a multidisciplinary team constituted of speech—language pathologists and neurologists. 

## 2. Methods

The ethical approval was obtained from the Institutional Review Board at PNU [approval no. 20-0386] in Riyadh, Saudi Arabia. The informed consent was also obtained from the patient prior to the publication of this case report. 

### Case Description

A 55-year-old male patient with a known medical history of diabetes mellitus type 2, treated lymphoma with no recurrence for 5 years, and a history of minor stroke with no residual presented to another hospital emergency department for a history of sudden left-sided facial asymmetry with mouth deviation to the left side with no other neurological symptoms. He underwent a Computerized Tomography (CT) scan of the brain, which showed focal hypodensity of the posterior limb right internal capsule indicative of an acute infarct and an old lacunar infarction of the small left basal ganglia. The patient was prescribed dual antiplatelet (aspirin and clopidogrel) therapy and discharged. 

One week later, the patient’s condition had worsened with increased facial weakness and new symptoms comprising left-sided weakness of both limbs, slurred speech, and difficulty swallowing. He was admitted to our hospital for workup for a suspected ischemic stroke. On examination, the patient had left-sided lower motor neuron facial palsy, decreased motor power on the left side, and diminished gag reflexes, more on the left side, that required a nasogastric tube (NGT) for feeding and the avoidance of aspiration pneumonia. A subsequent assessment included a brain CT scan, which showed multiple new scattered infarctions of indeterminate ages. A brain magnetic resonance imaging (MRI) confirmed bilateral diffuse scattered foci (bilateral central semiovale, thalamus, and internal capsule), with restricted diffusion, more on the right side. In addition, it revealed foci in the left frontal, right and left parietal subcortical white matter, brain stem, and pons, right middle cerebral and cerebellar peduncles and cerebellum, suggestive of multiple foci of acute infarctions, most likely embolic in nature. The Computerized Tomography angiography of the circle of Willis and the aorta showed no stenosis, thrombi, or aneurysms. A transthoracic Echocardiogram (TTE) and transesophageal echocardiogram (TEE) reveal no thrombi or shunts. No arrhythmia was detected in 48H Holter monitoring. Thrombosis workup, including CT Chest, abdomen, and pelvis (CT), for lymphoma recurrence was negative. The treatment with clopidogrel was continued and aspirin was discontinued. 

**Figure 1:**
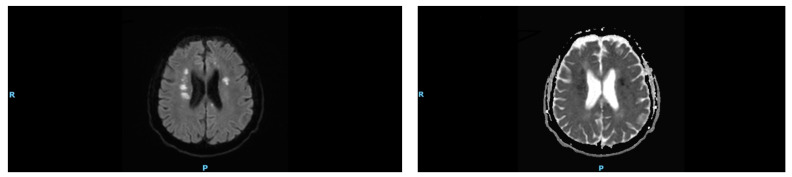
MRI brain showing high signal change in the diffusion-weighted sequence (**right**), with corresponding hypointensity in the periventricular and left frontal subcortical area in an apparent diffusion coefficient (ADC) map (**left**).

The MRI brain diffusion-weighted image revealed bilateral diffuse scattered foci of restricted diffusion of variable sizes, mainly small, with more on the right side innovating the bilateral central semi ovaleas as well the left frontal brain stem, pons, and cerebellum (Figure 1).

As the patient had difficulty swallowing, he was referred to a speech and language pathologist for a swallowing assessment. This referral happened 1 week after admission to our hospital. As part of the swallowing assessment, an oral motor examination (OME) was conducted to evaluate the following structures and their functions: mandible, lips, tongue, jaw, soft palate, palatal and pharyngeal reflexes, volitional cough, and quality of voice. During the assessment, pressure was applied to the patient’s lips and tongue using the rounded end of a cotton bud and the patient was asked to respond when he detected the touch sensation. The OME revealed no evidence of motor or sensory facial deficits or oral cavity deficits. However, a negative gag reflex on the left side was observed, with a strong voluntary cough. The patient’s tongue and jaw motion were as normal, with a good symmetrical movement of the velum, and he performed all tasks correctly upon command during the OME.

A bedside swallowing assessment proceeded after this examination. In this assessment, the patient was provided with a swallowing trial of four different volumes (i.e., 1, 3, 5, and 10 mL) of thin liquid followed by thick liquid consistency and, as an attempt, the sour was tried once with thin liquid. Then, a teaspoon and tablespoon of a puree consistency was also tried. With different volumes and consistencies and presenting the sour for only one attempt, the patient showed pocketing, lingual searching motion, uncoordinated lip and tongue movements, and anterior spillage. When the patient was provided with a swallowing, he appeared to struggle, as evidenced by tapping hand movements and head movement, showed pocketing, lingual searching motion, and uncoordinated lip and tongue movements, therefore suction was needed to remove the boluses that were tried. Despite performing well in the OME, his performance in the bedside swallowing assessment was poor and he struggled to swallow when bolus was presented in his mouth. 

A videofluoroscopic swallowing study (VFSS) revealed significant oral dysfunction, characterized by pocketing lasting for longer than 5 min and uncoordinated tongue manipulation movement with 1 mL of thin liquid. The patient experienced difficulty in initiating oral transfer to the pharynx when attempting to shift the bolus to the pharyngeal stage by moving his head sideways to the right and then left or back. Then, a 3 mL of thin liquid was attempted, but the same issue occurred. Therefore, it was not possible to perform a proper assessment of the pharyngeal stage during the VFSS.

Given these features, the swallowing difficulty noted in the voluntary control of oral preparatory and transfer stages and the absence of motor or sensory deficits, a provisional diagnosis of swallowing apraxia was produced. The focus of the provided management was on adjustments to facilitate eating without commands. The patient was orally administered frequent small flavored boluses (i.e., 1–2 mL, mostly sour) throughout the day. However, his nutritional intake was primarily through the NGT. The swallowing team followed him as an inpatient five times per week for 3 weeks and his performance fluctuated throughout this time. In the last few days prior to his transfer to another facility, he would occasionally swallow immediately, with no evident difficulty and no pocketing or coughing.

A fiberoptic endoscopic evaluation of a swallowing (FEES) study was conducted after three weeks to re-assess the patient. The FEES study was selected to enable a comprehensive assessment of swallowing, with each of the 1-, 3-, 5-, and 10-mL volume of thin and thick liquids and 1 teaspoon and tablespoon of puree, while avoiding radiation exposure. The FEES study lasted approximately 20 min. During this time, the patient was not provided with any commands and there were no distractions. Of note, no aspiration was noted when he swallowed his saliva of his own accord and with no provided commands. Although he demonstrated an improvement in the oral stage, with less pocketing, uncoordinated lingual movement, with searching motions, resulting in premature spillage, an aspiration was observed when he was instructed to swallow. Unique aspiration characteristics observed in the present case included inconsistent aspiration, which was unrelated to a specific consistency, amount of liquid/food, or manner of eating. Unfortunately, we were unable to continue our rehabilitation efforts, as the patient was moved to another hospital 3 weeks postadmission. 

## 3. Discussion 

In this case report, we describe an interesting case of swallowing apraxia after an ischemic stroke and case management by a multidisciplinary team constituted of speech and language pathologists and neurologists. During the bedside evaluation, the patient showed pocketing, lingual searching motions, uncoordinated lip and tongue movements, and anterior spillage. He struggled when initiating oral transfer to the pharynx and, as a consequence, he could not swallow. The OME revealed no apparent motor or sensory facial deficits or oral cavity deficits and swallowing difficulty was observed only when he had food in his mouth, with an excessive abnormal delay in the oral phase. There was no decline in cognitive function and the patient was oriented, able to follow simple and complex commands, and answer all questions in an intelligible manner, albeit with mildly slurred speech. 

Lingual discoordination could explain the delay in the oral phase of swallowing the present case. However, this was not considered the cause in our case. In lingual discoordination, the delay during the oral phase lasts only 2–3 s [9]. Our patient showed grossly abnormal delay. Although buccofacial apraxia (also known as oral apraxia) can affect the oral phase of swallowing function in stroke patients, this was not considered as a possible diagnosis in our patient, as he had normal lips and tongue movement when food was not present in his mouth and was able to produce oral postures on command, with normal involuntary productions [7,10]. The unique characteristics of dysphagia in the present case closely matched those of an apraxia subtype, swallowing apraxia. It is well known that swallowing apraxia is associated with strokes occurring in periventricular white matter and the left anterior cortex [6]. In our patient, acute infarctions affected these two critical areas of the brain, as shown by brain MRI (Figure 1).

To diagnose this case, we performed a bedside swallowing assessment, VFSS, and FEES study. The VFSS provided significant results related to the patient’s lingual movement. However, as the patient did not swallow during this assessment, we were unable to exclude pharyngeal dysphagia. In addition to avoiding radiation exposure, our aim in conducting the FEES study was to assess a full meal, without time constraints. This evaluation was also unable to rule out pharyngeal dysphagia because of the patient’s inconsistent aspiration. 

As our patient aspirated when he was instructed to swallow, it is logical to assume that the involuntary component of swallowing was intact. Of note, when not provided with any instructions, he swallowed normally, without aspiration. Both the VFSS and FEES study were important when diagnosing the patient’s swallowing condition. However, we recommend that clinicians use their clinical judgment when deciding which assessment tool to use for patients who present with swallowing difficulties. 

We currently lack adequate information on the best way to manage swallowing apraxia and on which treatment options are the most effective. In a case study, Yun et al. [7] discussed compensatory swallowing techniques to help patients pass food from the oral stage to the pharynx. The authors did not recommend neck extensions as a compensatory swallowing technique because it is difficult to assess the function of the pharyngeal stage during neck extension and they recommended the use of tube feeding to ensure adequate nutrition. Some studies have used transcranial direct current stimulation (tDCS) of the primary sensory and motor cortex in patients with swallowing apraxia caused by a stroke [11,12]. These studies found that the symptoms of swallowing apraxia significantly improved after a course of tDCS treatment. As the patient was transferred to another facility three weeks after admission to our hospital, we could not apply the method used in these studies. To improve the patient’s swallowing, we emphasized the importance of not instructing the patient to swallow and we frequently provided small, mostly sour-flavored, boluses of food. We found these techniques to be effective to some extent because the sour flavor helped to initiate the involuntary swallow reflex stimulation and therefore facilitated faster swallowing. Furthermore, in the absence of instructions, the patient struggled less when swallowing and immediately swallowed several times.

The brief time we were able to work with this patient (i.e., three weeks) affected our ability to fully assess these management techniques for swallowing dysfunction. As his performance in the various tests fluctuated throughout this time, with a slight improvement observed by the end of the 3 week-period, we recommended the continued use of an NGT for nutrition. In addition, we advised that the patient may require a percutaneous endoscopic gastrostomy tube in the long term if he continued to have difficulty swallowing and the oral stage of swallowing failed to improve.

## 4. Conclusions

The patient developed a swallowing dysfunction after experiencing an ischemic stroke that was compatible with swallowing apraxia. In the present case, we implemented two rehabilitation techniques, which resulted in some improvement in swallowing function. As swallowing rehabilitation commenced immediately after the onset of the patient’s swallowing difficulty, this may have played a role in his improvement. This report highlights the complexity of swallowing apraxia and contributes to an understanding of the identification and management of this poorly understood disorder. However, it should be noted that there is still a dearth of information on the effective management of swallowing apraxia. Therefore, we strongly recommend further studies on this topic.
